# High-Resolution Gene Flow Model for Assessing Environmental Impacts of Transgene Escape Based on Biological Parameters and Wind Speed

**DOI:** 10.1371/journal.pone.0149563

**Published:** 2016-03-09

**Authors:** Lei Wang, Patsy Haccou, Bao-Rong Lu

**Affiliations:** 1 Ministry of Education Key Laboratory for Biodiversity and Ecological Engineering, Department of Ecology and Evolutionary Biology, Fudan University, Handan Road 220, Shanghai 200433, China; 2 Leiden University College The Hague, P.O. Box 13228, 2501 EE The Hague, the Netherlands; Central China Normal University, CHINA

## Abstract

Environmental impacts caused by transgene flow from genetically engineered (GE) crops to their wild relatives mediated by pollination are longstanding biosafety concerns worldwide. Mathematical modeling provides a useful tool for estimating frequencies of pollen-mediated gene flow (PMGF) that are critical for assessing such environmental impacts. However, most PMGF models are impractical for this purpose because their parameterization requires actual data from field experiments. In addition, most of these models are usually too general and ignored the important biological characteristics of concerned plant species; and therefore cannot provide accurate prediction for PMGF frequencies. It is necessary to develop more accurate PMGF models based on biological and climatic parameters that can be easily measured *in situ*. Here, we present a quasi-mechanistic PMGF model that only requires the input of biological and wind speed parameters without actual data from field experiments. Validation of the quasi-mechanistic model based on five sets of published data from field experiments showed significant correlations between the model-simulated and field experimental-generated PMGF frequencies. These results suggest accurate prediction for PMGF frequencies using this model, provided that the necessary biological parameters and wind speed data are available. This model can largely facilitate the assessment and management of environmental impacts caused by transgene flow, such as determining transgene flow frequencies at a particular spatial distance, and establishing spatial isolation between a GE crop and its coexisting non-GE counterparts and wild relatives.

## Introduction

The potential environmental impact caused by transgene flow from a genetically engineered (GE) crop to its non-GE counterparts (crop-to-crop) and to wild relatives (crop-to-wild) through pollen-mediated gene flow (PMGF) has aroused great biosafety concerns worldwide, as a result of the extensive global cultivation of GE crops [1,2]. Field surveys and experimental studies suggest the great likelihood of crop-to-crop and crop-to-wild PMGF for world major crops [1,3,4]. For example, crop-to-crop PMGF is reported in a number of crop species, such as rice [5,6], wheat [7], maize [8], oilseed rape [9], soybean [10], potato [11], and cotton [12]. Likewise, crop-to-wild PMGF is also documented in wild/weedy relative species of rice [13,14], wheat [15], maize [16], sorghum [17], oilseed rape [18], sugar beet [19], soybean [20], and potato [21]. All these results indicate the potential of transgene escape to non-GE crops and wild relative species of the crops through PMGF, from which the undesired environmental impacts become a great concern with the commercialization of GE crops worldwide. Thus, the accurate measurement and prediction of PMGF frequencies becomes the key to assessing and managing the environmental impact from transgene escape, particularly when crop wild relatives are involved in such (trans)gene flow [1–4].

Usually, two approaches are applied to estimate PMGF frequency: conducting field experiments and mathematical modelling. The estimate of PMGF frequencies based on a field experiment is a common practice for environmental biosafety assessment [8,9,22,23,24]. In fact, PMGF data generated from field experiments have played an important role in assessing environmental consequences of transgene flow prior to the commercial production of GE crops. However, PMGF data collected from a particular field experiment have a limited use because these data cannot be applied to accurately assess PMGF of a crop species under diverse environmental conditions and in different cases. It is therefore difficult, if not impossible, to develop a general guideline that is useful for predicting PMGF frequencies of a crop species, based only on a limited number of field experiments at a limited number of sites [25,26]. In addition, to generate PMGF data from actual field experiments is time consuming and very expensive.

Mathematical modelling provides an important alternative method for estimating PMGF frequencies, which can overcome the constraints of merely relying on field experimental data. Consequently, many models are developed and used to predict PMGF frequencies for different crops [25–29]. These PMGF models can be roughly categorized into three types: (1) the mechanistic, (2) empirical, and (3) quasi-mechanistic models, based on the various calculation methods and parameters included for modeling. The early PMGF prediction used the mechanistic modeling that was essentially based on the physical dispersion of particles in the atmosphere [30,31]. Mechanistic modeling can be used to estimate PMGF frequencies without field experiments. However, this type of models is entirely based on the climatic parameters, without the consideration of any biological factors that are important for PMGF prediction, such as, the characteristics of donor’s pollen grains, the outcrossing rate of a pollen recipient, and cross-compatibility between a pollen donor and recipient of the involved plant species. Therefore, mechanistic models are too general and cannot accurately predict the PMGF frequency for a particular plant species, which may not be useful for assessing environmental impacts caused by transgene flow [32].

The empirical modeling includes actual data collected from field experiments, based on which a consensus level of PMGF is calculated for a particular pair of plant species (e.g., crop *versus* wild relative species) or populations under different environmental conditions [28,33,34]. The consensus level (or model) is then used to estimate the PMGF frequencies of that particular pair of plant species/populations. Empirical models can provide a more accurate prediction of PMGF, compared to the mechanistic models. However, all parameters necessary for empirical models must be generated based on a large number of PMGF field experiments [28,33].

Combined the merits of the previous two types of models, the quasi-mechanistic modeling can be used to predict the PMGF frequencies with an improved accuracy and a less number of field experiments [25,26,27,35]. This type of modeling includes the climatic parameters that can be directly measured *in situ*. However, this type of modeling still requires some critical parameters, such as the gene flow coefficient (GFC) [25] and synthetic parameter [27] that must be generated from the field experiments. The requirement of these parameters makes the PMGF modeling impractical to use and dependent of field experiments. Therefore, it is necessary to develop a more practical model that can predict gene flow frequencies more accurately but without data from actual PMGF field experiments.

To achieve the accurate description of PMGF, biological and climatic factors that determine the PMGF frequency should be considered in the modeling. Also, it should be more practical to predict gene flow frequencies only involving measurable parameters that are independent of any PMGF field experiment in the modeling. Rong et al. 2010 [26] developed a quasi-mechanistic model to estimate PMGF frequencies in rice, which included both biological (e.g., outcrossing rate of a pollen recipient, and cross compatibility between a pollen donor and recipient) and climatic (e.g., wind speed and humidity) factors. The model by Rong et al. 2010 [26] attempted to estimate PMGF frequencies more reliably than those only including climatic parameters. However, the model included a decay parameter of an exponential function—a *β* value that describes the pollen dispersal pattern, for which one needs to conduct field experiments to generate relevant data. In addition, the *β* values generated only from a few field experiments may not represent the decay parameter for all environmental conditions. Therefore, this quasi-mechanistic model [26] still has its constraint to estimate PMGF frequencies.

To overcome this constraint, we adopted the inverse Gaussian function [36] to replace the exponential dispersal function of the quasi-mechanistic model [26]. Through this replacement, we can avoid the inclusion of the experiment-dependent *β* values for PMGF modeling, and all the necessary parameters can be measured directly from the target environment. Here, we report the establishment of a modified quasi-mechanistic PMGF model using the inverse Gaussian function. The accuracy of the developed model was validated by comparing its simulation results with five sets of published PMGF data from field experiments. The modified model can provide a useful tool to assess the environmental impact of transgene flow and design suitable biosafety management strategies by accurately estimating PMGF frequencies without field experiments.

## Methods

### Procedure of model establishment

To establish a PMGF model, we used the inverse Gaussian function (Eq 1) by Katul et al. 2005 [36], which describes the wind dispersal pattern of small particles.

$$f\left(x\right)=\sqrt{\frac{\lambda }{2\pi x^{3}}}\text{ exp}\left(-\frac{\lambda {\left(x-\mu \right)}^{2}}{2\mu^{2}x}\right),$$ (1)

**Fig 1:**
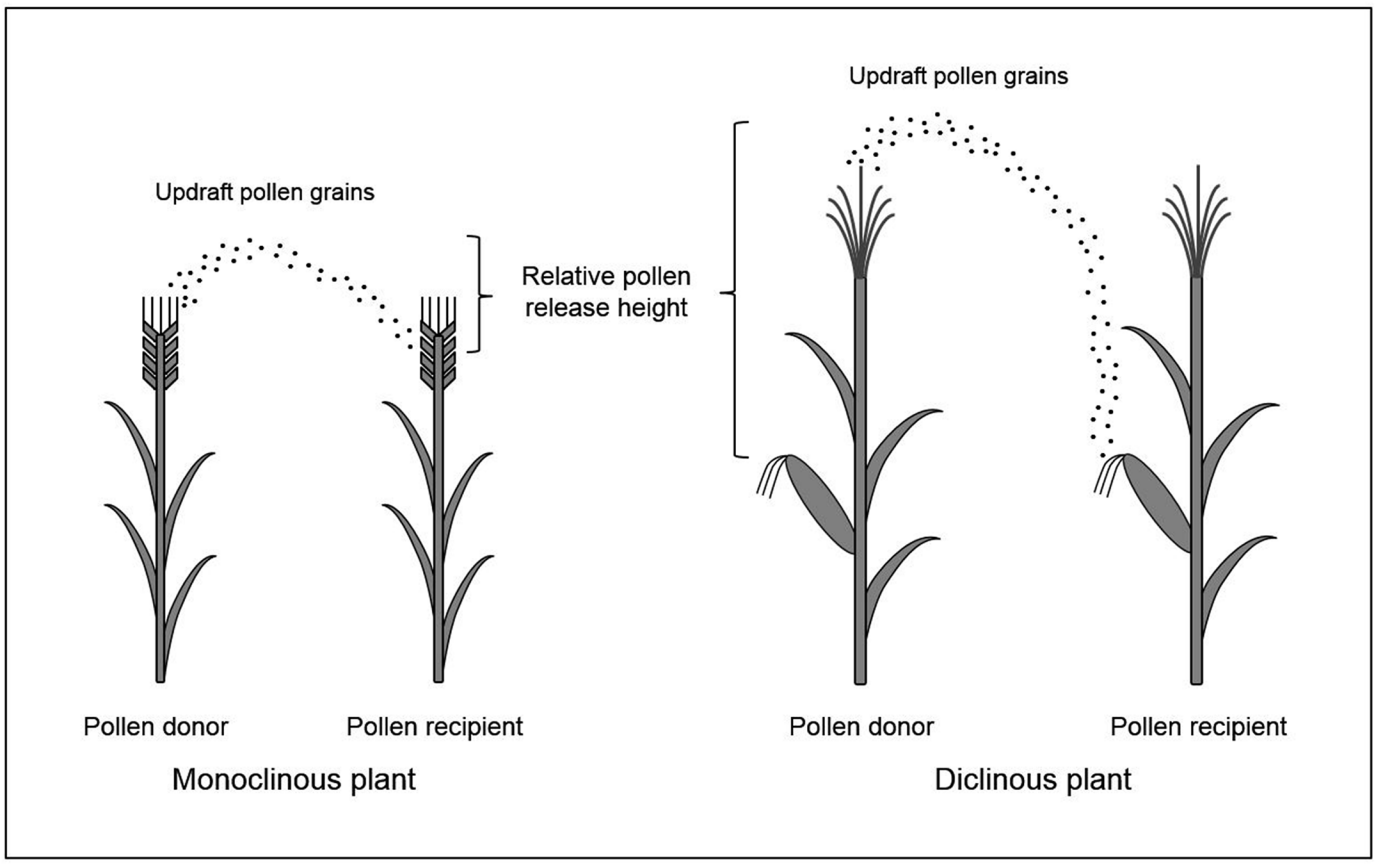
The illustration of relative pollen release height between a pollen donor and recipient under the consideration of updraft pollen grains. The left panel indicates the relative pollen release height of monoclinous plants and the right panel indicates the relative pollen release height of diclinous plants.

This function was used to predict the dispersal of released seeds, including the tiny ones such as *Senecio jacobaea* (~2 mm) and *Solidago rigida* (~3 mm) [36] from the plants onto the ground. In our case, this function is used to predict PMGF, in which the updraft pollen grains disperse from donors’ flowers to recipients’ flowers for monoclinous plants, and from male flowers (donors) to female flowers (recipients) for diclinous plants (Fig 1). Consequently, we made a slight modification to calculate the two key parameters included in the inverse Gaussian function of Katul et al. 2005 [36]: the scale parameter *λ* and the location parameter *μ*. As indicated by Katul et al. 2005 [36], the two parameters were estimated by the release height, wind speed, and deposition velocity. Here, we estimate the relative pollen release height, which is defined as the height between the position of updraft pollen grains of donor’s flowers and the position of recipient’s flowers for monoclinous plants (Fig 1, left panel), and the height between the position of updraft pollen grains of donor’s male flowers and the position of recipient’s female flowers for diclinous plants (Fig 1, right panel). Therefore, the equations (5b) and (9) of Katul et al. 2005 [36] to calculate *λ* and *μ* were modified as follows:

$$\lambda =h\bar{U}/2\kappa \sigma_{w},\;\;\mu =h\bar{U}/V_{t},$$ (2)

where *h* is the relative pollen release height (see Fig 1 for details); *Ū* is the average wind speed; *κ* is the von Karman’s constant (determined as 0.4) [36,37]; *σ*_*w*_ is the standard deviation of vertical wind speed; and *V*_*t*_ is the deposition velocity of pollen grains.

The wind speed (*Ū*) can be directly measured, and the standard deviation of vertical wind speed (*σ*_*w*_) is approximately equal to 1.1 × u_*_ [36]. Let *Ū* equals to the wind speed at the plant height, then u_*_ is calculated using the following equation [25,37]:

$$\bar{U}=\frac{u_{*}}{\kappa }\text{ln}\left(\frac{H-d}{z_{0}}\right),$$ (3)

where *H* is the plant height; *d* is the zero-plane displacement, which was commonly determined as 0.63 × *H* [25]; and z_0_ is the roughness length, which was determined as 0.13 × *H* [37].

The deposition velocity of pollen grains (*V*_*t*_) can either be obtained from previous studies or estimated by Stoke’s Law as follows [38,39]:

$$V_{t}=\frac{d_{p}{}^{2}g\left(\rho_{p}-\rho_{a}\right)}{18\eta },$$ (4)

where *d*_*p*_ is the diameter of a pollen grain (m); *g* is the gravitational acceleration (9.8 m s^−2^); *ρ*_*p*_ is the density of a pollen grain (kg m^−3^); *ρ*_*a*_ is the air density, which is equal to 1.178 kg m^−3^ under usual conditions; and *η* is the dynamic viscosity of air (~1.8×10^−5^ kg m^−1^ s^−1^) [40].

Through the above modification, we can describe the pollen dispersal pattern at different distances using the inverse Gaussian function which is based on the measurable biological and wind speed parameters. Therefore, we can establish a new model to predict PMGF frequencies using the inverse Gaussian function to replace the negative exponential function in the equation (7) developed by Rong et al. 2010 [26].

### Model validation

We used five sets of published data representing five major crop species (rice, wheat, maize, oilseed rape, and mustard) from field experiments [41–45] to test the accuracy of our modified PMGF model. The first set of data included PMGF frequencies generated from a transgenic herbicide-resistant rice (*Oryza sativa*) line to a wild rice (*O*. *rufipogon*) strain [41]. The second set of data included PMGF frequencies from a blue-grained wheat variety (*Triticum aestivum*) to a common wheat variety [42]. The third set of data included PMGF frequencies from a transgenic herbicide-resistant maize line (*Zea mays*) to its non-transgenic counterpart [43]. The fourth set of data included PMGF frequencies from a transgenic herbicide-tolerant oilseed rape line (*Brassica napus*) to a non-transgenic line [44]. The fifth set of data included PMGF frequencies from a transgenic canola (*Brassica napus*, AACC genomes) line resistant to herbicide (glyphosate) to its relative mustard (*B*. *juncea*, AABB genomes) for assessing PMGF between different species with divergent genomes [45].

All the parameters used for the model validation were listed in Table 1. Among these, the relative pollen release height was estimated as 0.5 m for the monoclinous wheat and oilseed rape assuming the updraft height of pollen grains from donor’s flowers is 0.5 m, using the updraft height of pollen grains of rice as a reference [46], because the hermaphrodite flowers of donors and recipients in these cases (wheat and *Brassica* species) are nearly at the same height [42,44,45]. The relative pollen release height was estimated as 0.2 m for the crop-to-wild rice gene flow, because the recipient’s flowers (wild rice) were ~0.3 m taller than the donor’s flowers (cultivated rice) [41]. The relative pollen release height was estimated as 1.5 m for the diclinous maize case, because the male flowers of maize were ~1.0 m higher than their female flowers [43].

**Table 1:** Detail information of the five sets of gene flow data from experimental fields used for the validation of the modified model.

Type of data	Relative pollen release height *h* (m)	Deposition velocity *V*_*t*_ (m s^−1^)	Outcrossing rate *t*_*B*_ (%)	Cross compatibility *δ*_*AB*_ (%)	Wind speed *Ū* (m s^−1^)	Donor field length *b* (m)	Field space *R* (m)
Crop-to-wild gene flow in rice	0.20	0.08	18.00 (GZ)* 24.00 (SY)*	70	1.8 (GZ) 2.6 (SY)	13 (GZ) 20 (SY)	0
Crop-to-crop gene flow in wheat	0.50	0.11	0.22 (year 2000) 0.10 (year 2001)	100	4.72	50	0
Crop-to-crop gene flow in maize	1.50	0.32	50.00	100	2.36 (year 2006) 1.92 (year 2007)	50	0
Crop-to-crop gene flow in oilseed rape	0.50	0.03	1.7	100	2.05	100	0
Canola-to-mustard gene flow	0.50	0.03	15.0	0.97	3.0	400	0

* GZ indicates Guangzhou site in Guangdong province; SY indicates Sanya site in Hainan province.

The parameter of deposition velocity of pollen grains (*V*_*t*_) for the first and third sets of data (rice and maize cases) was obtained from the published data [47,48], and that for the second and fourth sets of data (wheat and oilseed rape case) was estimated based on the pollen diameters of rice (40–50 μm), wheat (48–57 μm), maize (80–100 μm), and oilseed rape (24–26 μm) [49–51]following Eq 4. In addition, for the parameter of cross compatibility (*δ*_*AB*_) between a pollen donor and recipient, we set *δ*_*AB*_ as 100% for the second, third, and fourth sets of data because their pollen donors and recipients are the same species. For the first set of data, we set *δ*_*AB*_ as 70% following the literature by Oka who estimated the cross compatibility between cultivated rice and wild *O*. *rufipogon* [52], because the cultivated rice was used as the pollen donors and wild rice as the pollen recipients [41]. For the fifth set of data, we set *t*_*B*_ as 15.0% according to previous studies [53–55] and calculated *δ*_*AB*_ from *t*_*B*_ and observed PMGF data, and the wind speed was set as a moderate value of 3 m s^−1^ because it was not provided in this experiment [45]. In simulating the first set of data using Eq 5, we replaced the parameter of the recipient pollen density (*D*_*B*_) by the adjusted recipient pollen density (*δ*_*AB*_×*D*_*B*_) where pollen competition between GE and non-GE rice lines was considered because the recipient field included both wild and non-GE rice [41].

The analyses of covariance and correlation were applied to validate the accuracy of the model. The analysis of covariance was conducted to test the consistency of slopes (*p*_*s*_ values) and the consistency of intercepts (*p*_*i*_ values) between PMGF frequencies obtained from the published field experiment (represented by empty circles) and model simulation (represented by curves) at different spatial distances. Correlation analysis was also conducted to test the goodness-of-fit between the published field experiment data and model simulation results (*r* values). All the statistical analyses were conducted using the software SPSS ver. 22.0 (SPSS, Chicago, Illinois, USA).

## Results

### Model

We replaced the exponential function in the equation (7) of the PMGF model by Rong et al. 2010 [26] with the adjusted inverse Gaussian function of Katul et al. [36]. As a result, a modified quasi-mechanistic PMGF model is established, and can be used to predict the gene flow frequency (*F*_*AB*_ (*x*)) from a donor (A) to a recipient (B) as follows:

$$F_{AB}\left(x\right)=t_{B}\frac{\delta_{AB}D_{A}}{\delta_{AB}D_{A}+D_{B}}=t_{B}\frac{\delta_{AB}\left(\varphi \left(x+b\right)-\varphi \left(x\right)\right)}{\delta_{AB}\left(\varphi \left(x+b\right)-\varphi \left(x\right)\right)+\varphi \left(x-R\right)},$$ (5)

where *t*_*B*_ is the outcrossing rate of a recipient plant; *x* is the spatial distance from the edge of a donor field to the measured points of recipient fields; *b* is the length of the donor field; *R* is the spatial distance between donor and recipient fields (see Fig 1 in Rong et al. 2010 [26]); and *φ*(*x*) is the cumulative distribution function of the inverse Gaussian function, which can be calculated as follows [56]:

$$\varphi \left(x\right)=\int_{0}^{x}f\left(y\right)dy=\Phi \left(\sqrt{\frac{\lambda }{x}}\left(\frac{x}{\mu }-1\right)\right)+\text{exp}\left(\frac{2\lambda }{\mu }\right)\Phi \left(-\sqrt{\frac{\lambda }{x}}\left(\frac{x}{\mu }+1\right)\right),$$ (6)

where the symbol Φ denotes the cumulative distribution function of the standard normal distribution.

Here, the modified quasi-mechanistic PMGF model (Eq 5) is used to calculate PMGF frequencies for the dataset collected from a parallel donor-to-recipient field design, such as in the case of rice-wild rice and canola-mustard gene flow [41,45]. However, we found that the model (Eq 5) can also be applied for dataset collected from the donor-surrounded-by-recipient field design such as in the cases of wheat, maize, and oilseed rape [42–44]. This indicates that the modified PMGF model can produce well-fitted predicting results for datasets from the two types of field experimental designs.

### Validation

**Fig 2:**
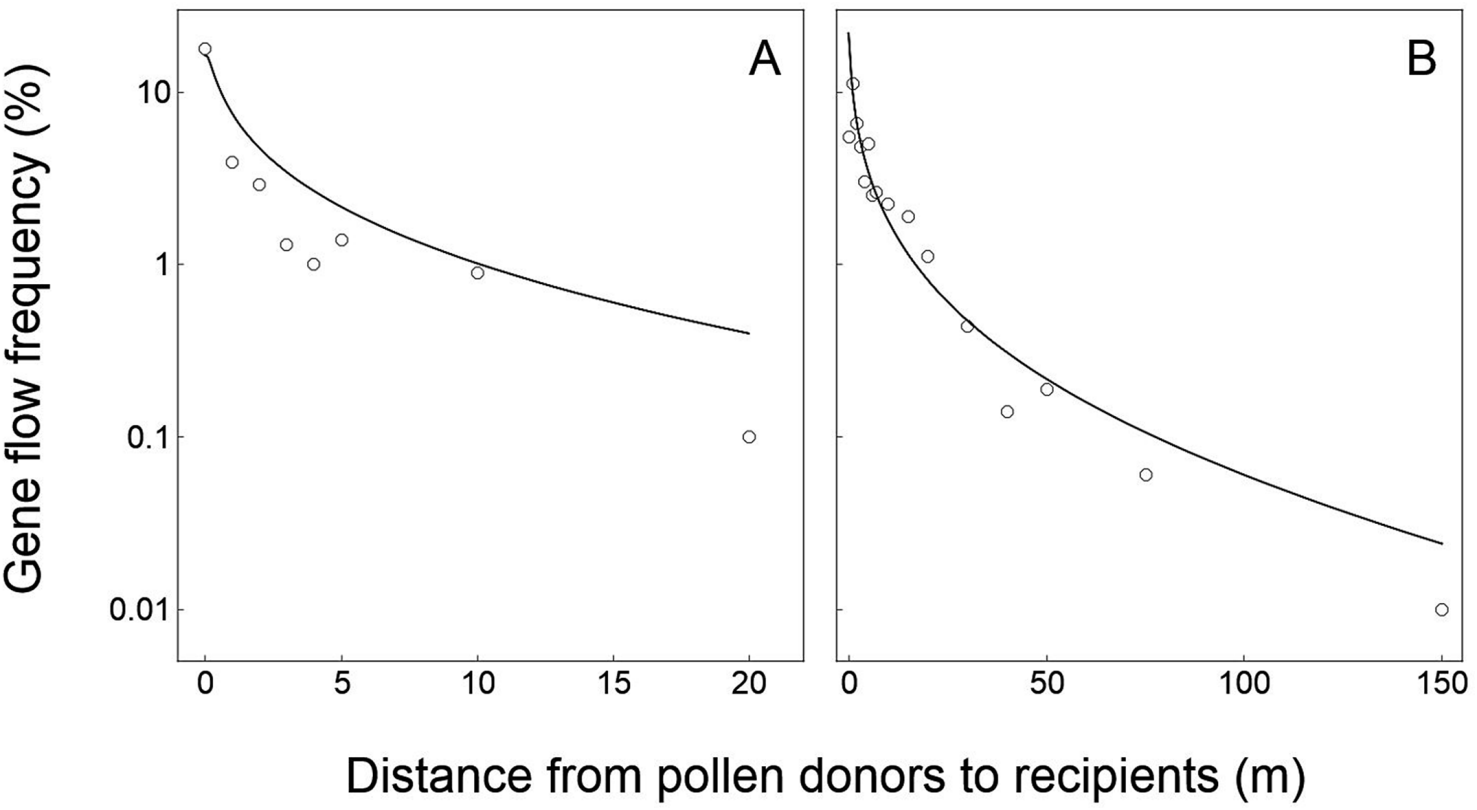
Relationships of crop-to-wild gene flow between field experiments (empty circles) and model-based simulation (solid curves) in rice (*Oryza sativa*, *O*. *rufipogon*) at various spatial distances. (A) Field experiment data from the site in Guangzhou. (B) Field experiment data from the site in Sanya [41]. Logarithmic coordinate axes (y-axis) were applied to indicate the gene flow frequencies.

**Fig 3:**
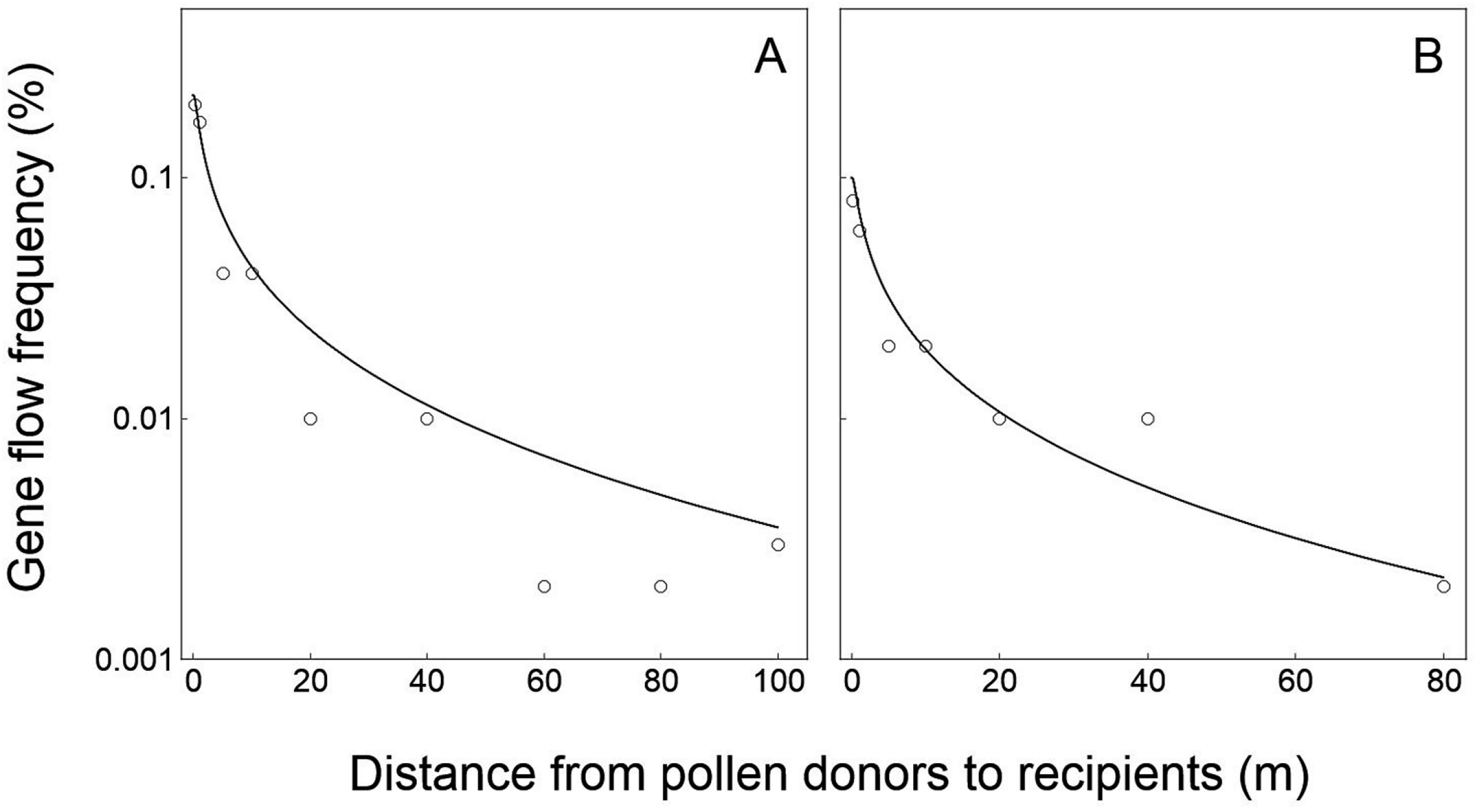
Relationships of crop-to-crop gene flow between field experiments (empty circles) and model-based simulation (solid curves) in wheat (*Triticum aestivum*) at various spatial distances. (A) Field experiment data collected in 2000. (B) Field experiment data collected in 2001 [42]. Logarithmic coordinate axes (y-axis) were applied to indicate the gene flow frequencies.

**Fig 4:**
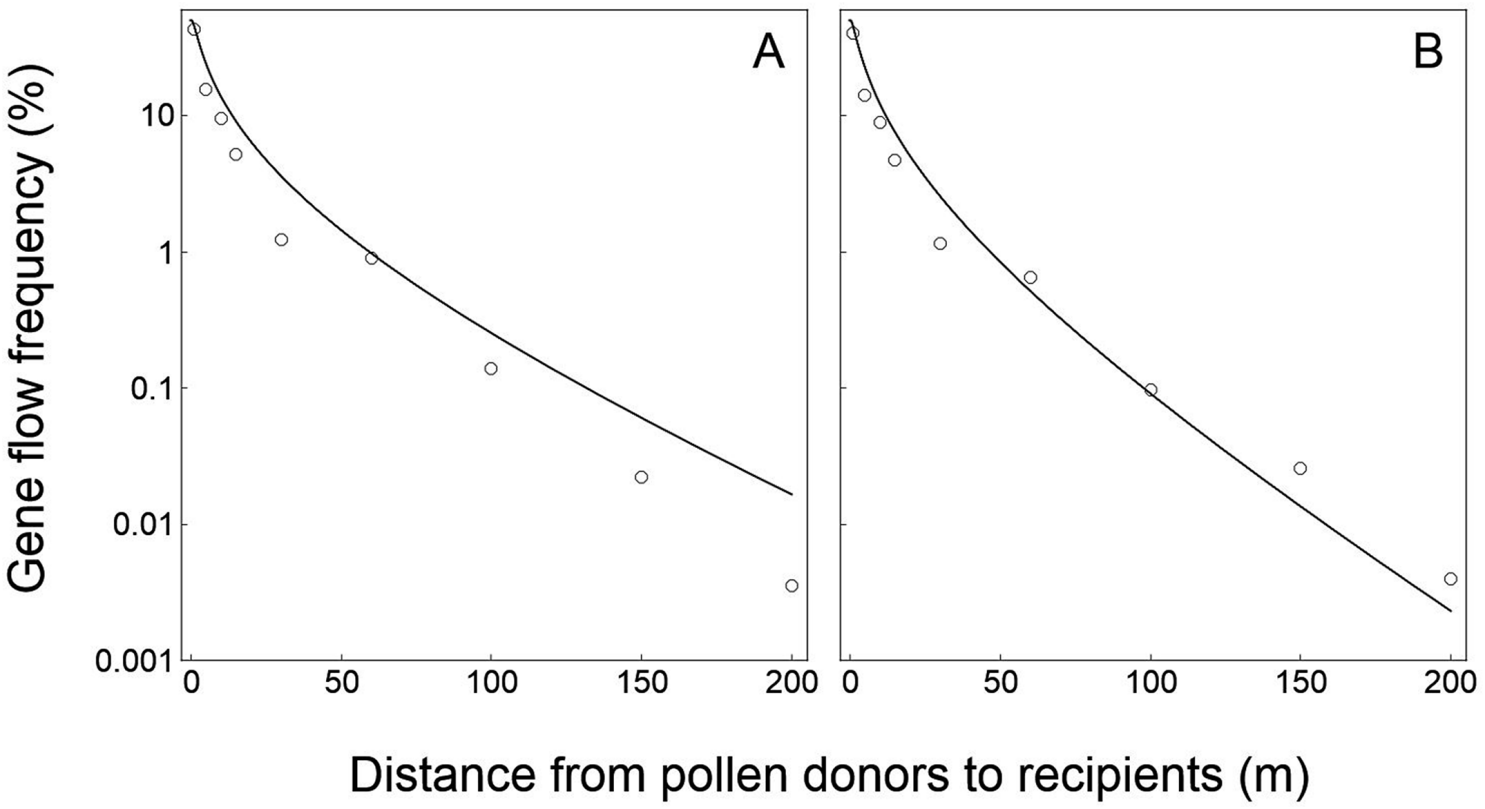
Relationships of crop-to-crop gene flow between field experiments (empty circles) and model-based simulation (solid curves) in maize (*Zea mays*) at various spatial distances. A. Field experiment data collected in 2006. (B) field experiment data collected in 2007 [43]. Logarithmic coordinate axes (y-axis) were applied to indicate the gene flow frequencies.

**Fig 5:**
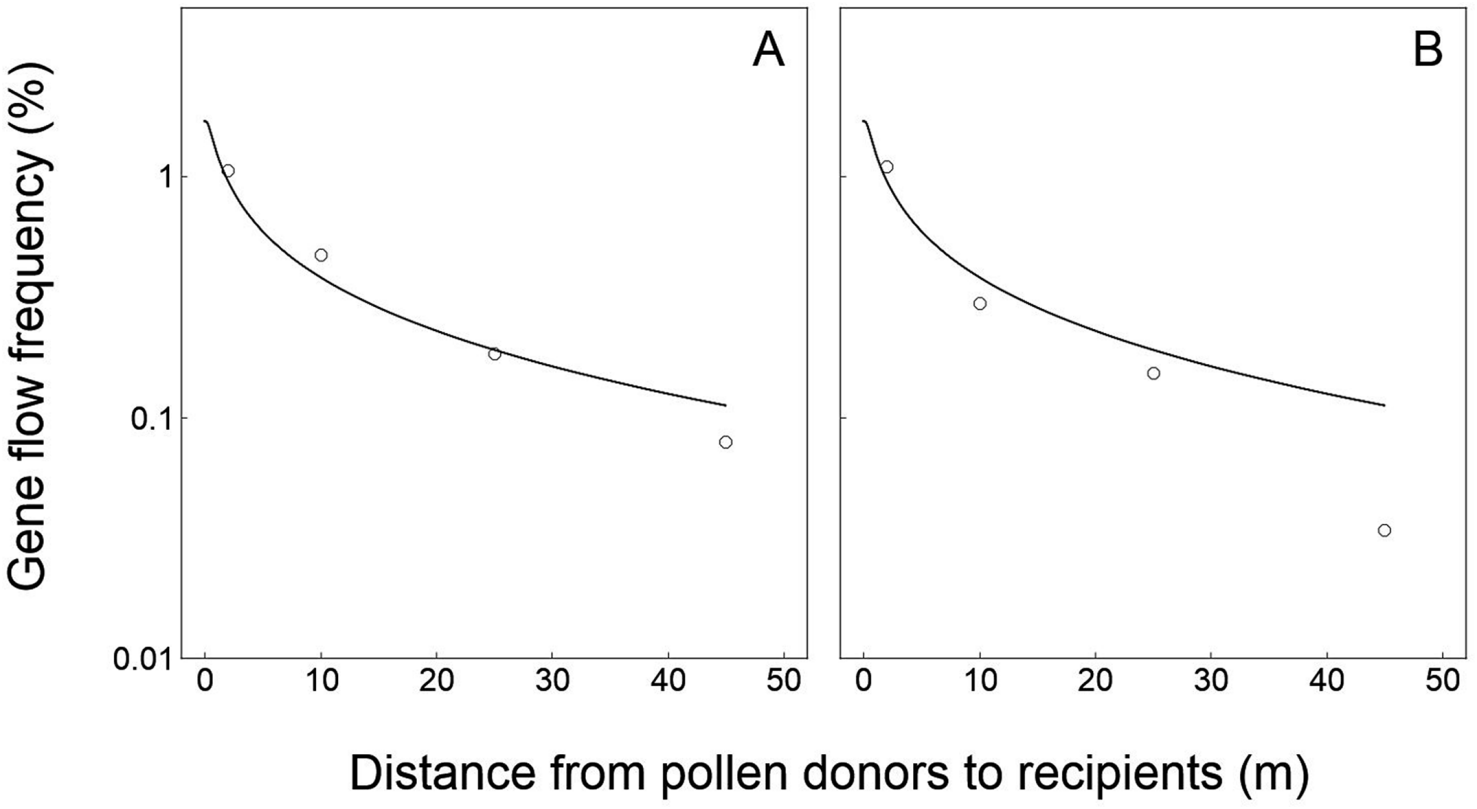
Relationships of crop-to-crop gene flow between field experiments (empty circles) and model-based simulation (solid curves) in oilseed rape (*Brassica napus*) at various spatial distances. (A) Field experiment data collected in 2008. (B) Field experiment data collected in 2009 [44]. Logarithmic coordinate axes (y-axis) were applied to indicate the gene flow frequencies.

**Fig 6:**
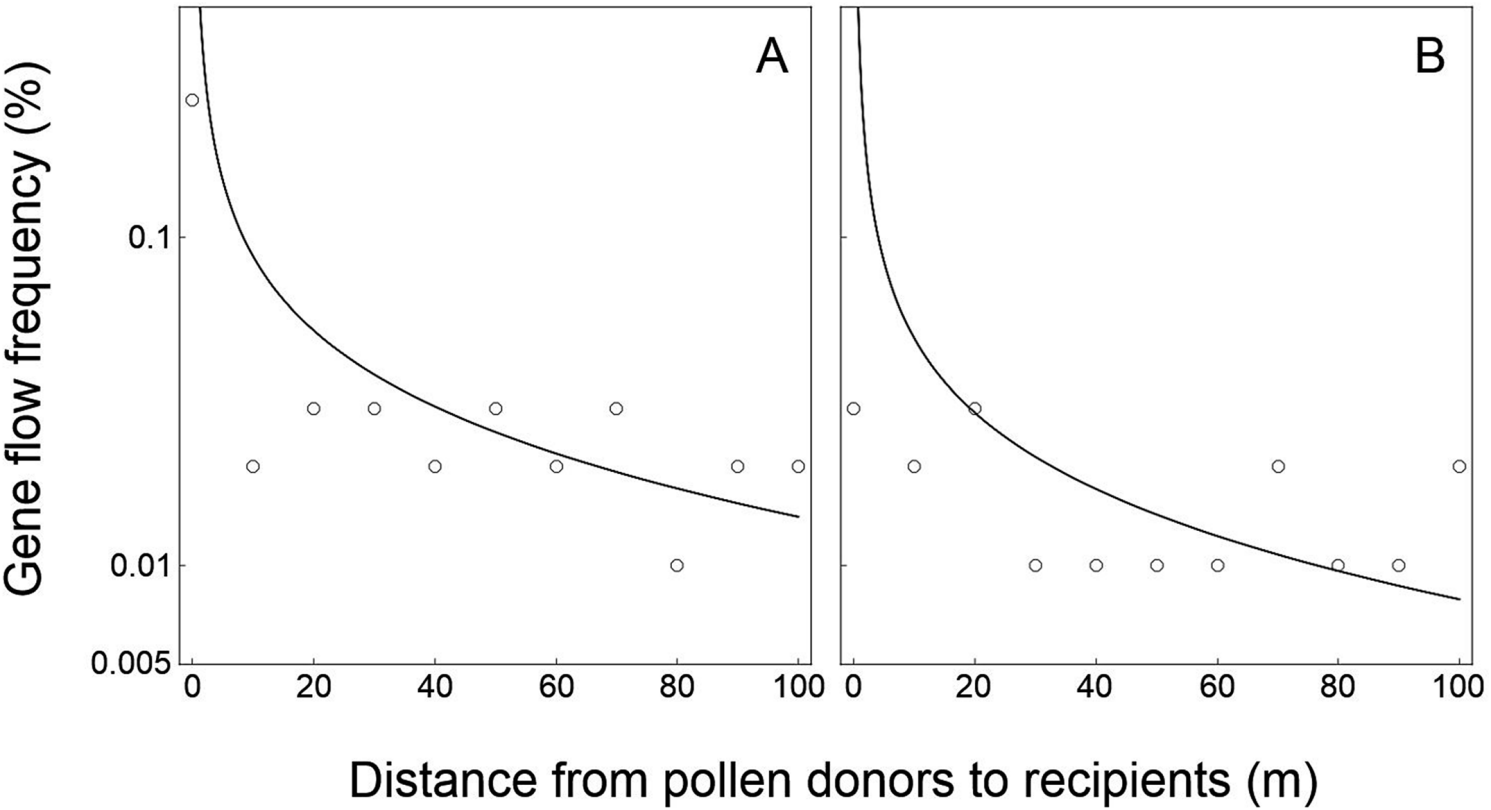
Relationships of canola (*Brassica napus*) to mustard (*B*. *juncea*) gene flow between field experiments (empty circles) and model-based simulation (solid curves) at various spatial distances. (A) Field experiment data collected at the site 1. (B) Field experiment data collected at the site 2 [45]. Logarithmic coordinate axes (y-axis) were applied to indicate the gene flow frequencies.

In general, the simulation results (represented by the curves) based on our modified PMGF model from this study fitted significantly well with the observed PMGF frequencies (represented by the circles) from the five sets of published data [41–45] generated from the field experiments for rice-wild rice, wheat-wheat, maize-maize, oilseed rape-oilseed rape, and canola-mustard gene flow (Figs 2–6).

For the rice data validation, the PMGF frequencies estimated based on the model simulation fitted perfectly with the frequencies obtained from the two independent crop-to-wild gene flow field experiments at the Guangzhou and Sanya sites (Fig 2). The *p*_*s*_ and *p*_*i*_ values between model-simulated and experiment-observed PMGF frequencies are 0.888 and 0.660 for data from the Guangzhou site, and 0.961 and 0.930 for data from the Sanya site, respectively. These values showed no significant differences (>0.05) in the slope (*p*_*s*_ value) and intercept (*p*_*i*_ value) of the curves representing PMGF frequencies at different spatial distances between the model-simulated and field-experimental results (Fig 2), indicating the similar PMGF trends of the model-simulated and experiment-observed data. In addition, the correlation coefficients (*r*) between the model-simulated and experiment-observed PMGF frequencies were 0.965 (*p*<0.001) and 0.979 (*p*<0.001) for data from the Guangzhou and Sanya sites, respectively. Significant positive correlation was observed between the PMGF frequencies obtained from the model-simulated and field-experimental results.

For the wheat data validation, the PMGF frequencies estimated based on the model simulation fitted perfectly with the frequencies obtained from the two independent crop-to-crop gene flow field experiments conducted in 2000 and 2001 (Fig 3). The *p*_*s*_ and *p*_*i*_ values between model-simulated and experiment-observed PMGF frequencies are 0.899 and 0.803 for the data from 2000 experiment, and 0.732 and 0.699 for the data from 2001 experiment, respectively. These values showed no significant differences (>0.05) in the slope (*p*_*s*_ value) and intercept (*p*_*i*_ value) of the curves representing PMGF frequencies at different spatial distances between the model-simulated and field-experimental results (Fig 3), suggesting the similar PMGF trends of the model-simulated and experiment-observed data. In addition, the correlation coefficients (*r*) between the model-simulated and experiment-observed PMGF frequencies were 0.989 (*p*<0.001) and 0.993 (*p*<0.001) for the data from 2000 and 2001 experiments, respectively. Significant positive correlation was observed between the PMGF frequencies obtained from the model-simulated and field-experimental results.

For the maize data validation, the PMGF frequencies estimated based on the model simulation fitted perfectly with the frequencies obtained from the two independent crop-to-crop gene flow field experiments conducted in 2006 and 2007 (Fig 4). The *p*_*s*_ and *p*_*i*_ values between model-simulated and experiment-observed PMGF frequencies are 0.741 and 0.664 for the data from 2006 experiment, and 0.728 and 0.667 for the data from 2007 experiment, respectively. These values showed no significant differences (>0.05) in the slope (*p*_*s*_ value) and intercept (*p*_*i*_ value) of the curves that represented PMGF frequencies at different spatial distances between the model-simulated and field-experimental results (Fig 4), indicating the similar PMGF trends of the model-simulated and experiment-observed data. In addition, the correlation coefficients (*r*) between the model-simulated and experiment-observed PMGF frequencies were 0.987 (*p*<0.001) and 0.990 (*p*<0.001) for the data from 2006 and 2007 experiments, respectively. Significant positive correlation was observed between the PMGF frequencies from the model-simulated and field-experimental results.

For the oilseed rape data validation, the PMGF frequencies estimated based on the model simulation fitted perfectly with the frequencies obtained from the two independent crop-to-crop gene flow field experiments conducted in 2008 and 2009 (Fig 5). The *p*_*s*_ and *p*_*i*_ values between model-simulated and experiment-observed PMGF frequencies are 0.779 and 0.830 for the data from 2008 experiment, and 0.805 and 0.939 for the data from 2009 experiment, respectively. These values showed no significant differences (>0.05) in the slope (*p*_*s*_ value) and intercept (*p*_*i*_ value) of the curves that represented PMGF frequencies at different spatial distances between the model-simulated and field-experimental results (Fig 5), indicating the similar PMGF trends of the model-simulated and experiment-observed data. In addition, the correlation coefficients (*r*) between the model-simulated and experiment-observed PMGF frequencies were both 0.996 (*p*<0.01) for the data from 2008 and 2009 experiments. Significant positive correlation was observed between the PMGF frequencies from the model-simulated and field-experimental results.

For the validation of canola-mustard gene flow data, the PMGF frequencies estimated based on the model simulation fitted well with the frequencies obtained from the two independent gene flow field experiments (Fig 6). The *p*_*s*_ and *p*_*i*_ values between model-simulated and experiment-observed PMGF frequencies are 0.116 and 0.343 for the data from the site 1 (Fig 6A), and 0.096 and 0.307 for the data from the site 2 (Fig 6B), respectively. These values showed no significant differences (>0.05) in the slope (*p*_*s*_ value) and intercept (*p*_*i*_ value) of the curves that represented PMGF frequencies at different spatial distances between the model-simulated and field-experimental results, indicating the similar PMGF trends of the model-simulated and experiment-observed data. In addition, the correlation coefficients (*r*) between the model-simulated and experiment-observed PMGF frequencies were 0.996 (*p*<0.001) and 0.563 (*p* = 0.071) for the data from the site 1 and site 2, respectively.

## Discussion

In this study, we established a new pollen-mediated gene flow (PMGF) model based on the quasi-mechanistic model of Rong et al. 2010 [26], by replacing the exponential function in the quasi-mechanistic model with the adjusted inverse Gaussian function of Katul et al. 2005 [36]. By such a modification, the new PMGF model can circumvent the input of a decay parameter (the *β* value) that must be generated from separate PMGF field experiments. Our modified model includes the following biological parameters: pollen diameter and relative pollen release height of a donor, outcrossing rate of a pollen recipient, cross compatibility between a pollen donor and recipient, spatial distance between donor and recipient fields, and the length (depth) of a donor field, in addition to the parameter of wind speed. All of these parameters can either be measured directly in the target locations or obtained from published studies/databases. In contrast to the model of Rong et al. 2010 [26], some climatic factors such as the temperature and relative humidity were not considered in our modified PMGF model, although they may affect PMGF frequencies [26,57,58,59]. This is because the relationship between the two factors and PMGF frequencies has not yet been accurately determined. Consequently, we neglected the temperature and relative humidity in our modified PMGF model. Once the relationship is clearly determined in the future, we can include the two parameters in the PMGF model. Thus, our modified PMGF model can be applied to accurately calculate/predict the gene flow frequencies of plant species mediated by pollen at different spatial distances under various environments, independent of PMGF field experiments. The new feature of our modified model makes the estimation of PMGF frequencies more practical and is easier to use, provided that the required biological parameters and wind speed data are available.

To validate the modified PMGF model, we compared the model-simulated PMGF pattern and the gene flow frequencies obtained from the field experiments. The results of model-simulated and experiment-generated PMGF showed a high level of goodness-of-fit, suggesting the high predicting power for gene flow frequencies using the modified PMGF model. The high probability (*p*_*s*_) and (*p*_*i*_) values (>0.05) indicate the consistency of the slopes and the intercepts between the PMGF frequencies obtained from the model simulation and the gene flow data from the five sets of field experiments. The high correlation coefficients (*r* values, >0.90) indicate the consistency of PMGF frequencies between the results obtained from the model simulation and the five sets of gene flow data, except for the site 2 of the canola-mustard PMGF experiment in the fifth set of data [45], which showed slightly lower *r* value (~0.56). Notably, the slightly weaker correlation between the model-predicted result and observed data from the site 2 is probably due to the unexpectedly low PMGF frequencies (0.03%) observed at the close distance (0 m), which is unusual in PMGF experiment. These results indicate the accuracy and effectiveness of the modified model for predicting PMGF frequencies. For example, based on the field experiment, Langhof et al. 2010 suggested the spatial isolation distance of 50 m between a GE maize and its non-GE counterpart with the size of 200 × 200 m^2^ because the frequency of transgene flow was reduced to <0.9% (the European standard for a gene flow frequency between co-existing GE and non-GE crops) at this isolation distance [60]. Similarly according to our model simulation, the predicted average PMGF frequency from a donor to a recipient maize field with the same situation was <0.7% (data is not shown) at the spatial distance of 50 m. The two examples showed considerable consistency between the field-experimental and model-simulated results. In addition, the gene flow frequencies of different wheat varieties generated from a field experiment were <0.1% at the spatial distance of 27 m [61], which was highly consistent with the frequencies generated from model simulation. All these indicate the usefulness of our modified model for the accurate prediction of PMGF frequencies.

Noticeably at the relatively long spatial distances, the model-predicted PMGF frequencies are slightly higher than the gene flow frequencies obtained from field experiments in some cases. This is probably due to the viability of pollen grains in the air, which can considerably influence the actual gene flow frequencies at the long spatial distance. Pollen grains of many plant species, particularly wind-pollination species, lose their viability quickly after being released to the air [62,63]. The number of viable pollen grains might be considerably reduced after a long-distance travel in the air. Therefore at the long spatial distance, the model-predicted PMGF frequency becomes greater than actually obtained gene flow frequencies from field experiments. Nevertheless, the predicted PMGF frequencies based on our modified model can provide useful information for assessing and monitoring environmental consequences caused by (trans)gene flow, particularly for the worst scenario assessment [26,64] and for establishing a safe isolation spatial distance between co-existing GE and non-GE crops [8,63].

With the rapid expansion and commercial production of GE crops over the world, transgene flow and its environmental impact has aroused increasing biosafety concerns worldwide [65]. The effective assessment and monitor of the environmental impact caused by transgene gene flow are important to guarantee the safe and sustainable application of GE crops. To estimate the frequency (exposure) of transgene flow is the critical and first step for assessing and monitoring the environmental impact [66,67]. Our modified PMGF model provides a practical tool to meet the objective of transgene flow estimation, particular for wind-pollinated plant species. This model can be applied to estimate the frequency of crop-to-crop and crop-to-wild transgene flow relatively accurately for target plant species in various locations, based only on the required biological parameters and wind speed data that can be easily obtained. The application of our modified PMGF model can make the prediction of transgene flow more effective and timely, because the modeling has excluded the expensive and time-consuming gene flow filed experiments to generate necessary parameters. In addition, the strategic design of a spatial isolation distance can be made between coexisting GE and non-GE crops based on the model predicted transgene flow frequencies to minimize the transgene “contamination” to a permitted threshold value [60,68]. Similarly, a proper spatial isolation distance can be established between a GE crop and its wild relative species to restrict transgene flow based on the model predicted result for reducing the potential environmental impact [69]. This can largely facilitate the biosafety assessment and management concerning the transgene flow and its environmental impact. Furthermore, the model-based prediction of gene flow can also be applied in the studies of evolutionary and invasive biology [70,71].

In conclusion, we established a PMGF model with a great advantage and potentially broader use for predicting pollen-mediated gene flow, provided that the desired biological parameters and wind speed data are available. This model retains the feature of accuracy of Rong et al. 2010 model [26], but has added a new feature that no longer requires the input of field experimental data to generate parameters as in previous models [26,28,35]. The validation results indicate the accuracy of the model prediction for gene flow of various plant species. Because this PMGF model is practical to use and free of field experiment, it can be broadly applied in assessing (trans)gene flow. Therefore, this PMGF model can be useful for biosafety assessment of potential environmental impact caused by transgene flow, in addition to addressing related questions in evolutionary biology.

## References

[1] Ellstrand NC (2003) Current knowledge of gene flow in plants: implications for transgene flow. Philosophical Transactions of the Royal Society of London Series B-Biological Sciences 358: 1163–1169. 10.1098/rstb.2003.1299 PMC1693201 12831483

[2] Felber F, Kozlowski G, Arrigo N, Guadagnuolo R (2007) Genetic and ecological consequences of transgene flow to the wild flora. Advances in Biochemical Engineering-Biotechnology 107: 173–205. 10.1007/10_2007_050 17522826

[3] Ellstrand NC, Meirmans P, Rong J, Bartsch D, Ghosh A, de Jong TJ, et al (2013) Introgression of crop alleles into wild or weedy populations. Annual Review of Ecology, Evolution, and Systematics 44: 325–345.

[4] Ellstrand NC, Hancock JF (1999) Gene flow and introgression from domesticated plants into their wild relatives. Annual Review of Ecology and Systematics 30: 539−563.

[5] Messeguer J, Fogher C, Guiderdoni E, Marfà V, Català MM, Baldi G, et al (2001) Field assessments of gene flow from transgenic to cultivated rice (Oryza sativa L.) using a herbicide resistance gene as tracer marker. Theoretical and Applied Genetics 103: 1151–1159.

[6] Rong J, Xia H, Zhu YY, Wang YY, Lu B-R (2004) Asymmetric gene flow between traditional and hybrid rice varieties (Oryza sativa) indicated by nuclear simple sequence repeats and implications for germplasm conservation. New Phytologist 163: 439–445. 10.1111/j.1469-8137.2004.01100.x 33873619

[7] Hanson BD, Mallory-Smith CA, Shafii B, Thill DC, Zemetra RS (2005) Pollen mediated gene flow from blue aleurone wheat to other wheat cultivars. Crop Science 45: 1610–1617.

[8] Messequer J, Pañas G, Ballester J, Bas M, Serra J, Salvia J, et al (2006) Pollen-mediated gene flow in maize in real situations of coexistence. Plant Biotechnology Journal 4: 633–645. 17309734 10.1111/j.1467-7652.2006.00207.x

[9] Weekes R, Deppe C, Allnutt T, Boffey C, Morgan D, Morgan S, et al (2005) Crop-to-crop gene flow using farm scale sites of oilseed rape (Brassica napus) in the UK. Transgenic Research 14: 749–759. 16245166 10.1007/s11248-005-0943-2

[10] Yoshimura Y, Matsuo K, Yasuda K (2006) Gene flow from GM glyphosate-tolerant to conventional soybeans under field conditions in Japan. Environmental Biosafety Research 5: 169–173. 17445512 10.1051/ebr:2007003

[11] Capurro MA, Camadro EL, Masuelli RW (2014) Gene flow between potato cultivars under experimental field conditions in Argentina. Potato Research 57: 111–122. 10.1111/j.1601-5223.2013.00018.x 24325306

[12] Zhang BH, Pan XP, Guo TL, Wang QL, Anderson TA (2005) Measuring gene flow in the cultivation of transgenic cotton (Gossypium hirsutum L.). Molecular Biotechnology 31: 11–20. 16118411 10.1385/MB:31:1:011

[13] Song ZP, Lu B-R, Zhu YG, Chen JK (2003) Gene flow from cultivated rice to the wild species *Oryza rufipogon* under experimental field conditions. New Phytologist 157: 657–665. 10.1046/j.1469-8137.2003.00699.x 33873418

[14] Chen LJ, Lee DS, Song ZP, Suh HS, Lu B-R (2004) Gene flow from cultivated rice (Oryza sativa) to its weedy and wild relatives. Annals of Botany 93: 67–73. 14602665 10.1093/aob/mch006 PMC4242260

[15] Guadagnuolo R, Savova-Bianchi D, Felber F (2001) Gene flow from wheat (Triticum aestivum L.) to jointed goatgrass (Aegilops cylindrica Host.), as revealed by RAPD and microsatellite markers. Theoretical and Applied Genetics 103: 1–8.

[16] Ellstrand NC, Garner LC, Hegde S, Guadagnuolo R, Blancas L (2007) Spontaneous hybridization between maize and teosinte. Journal of Heredity 98: 183–187. 17400586 10.1093/jhered/esm002

[17] Arriola PE, Ellstrand NC (1996) Crop-to-weed gene flow in the genus Sorghum (Poaceae): spontaneous interspecific hybridization between Johnsongrass, Sorghum halepense, and crop sorghum, S. bicolor. American Journal of Botany 83: 1153–1159.

[18] Ford CS, Allainguillaume J, Grilli-Chantler P, Cuccato G, Allender CJ, Wilkinson MJ (2006) Spontaneous gene flow from rapeseed (Brassica napus) to wild Brassica oleracea. Philosophical Transactions of the Royal Society of London Series B-Biological Sciences 273: 3111–3115. 10.1098/rspb.2006.3686 PMC1804201 17015343

[19] Andersen NS, Siegismund HR, Meyer V, Jørgensen RB (2005) Low level of gene flow from cultivated beets (Beta vulgaris L. ssp. vulgaris) into Danish populations of sea beet (Beta vulgaris L. ssp. maritima (L.) Arcangeli). Molecular Ecology 14: 1391–1405. 15813779 10.1111/j.1365-294X.2005.02490.x

[20] Kuroda Y, Kaga A, Tomooka N, Vaughan DA (2008) Gene flow and genetic structure of wild soybean (Glycine soja) in Japan. Crop Science 48: 1071–1079.

[21] Capurro MA, Camadro EL, Masuelli RW (2013) Pollen-mediated gene flow from a commercial potato cultivar to the wild relative *S*. *chacoense* Bitter under experimental field conditions in Argentina. Hereditas 150: 60–65. 10.1111/j.1601-5223.2013.00018.x 24325306

[22] Rong J, Song ZP, Su J, Xia H, Lu B-R, Wang F (2005) Low frequency of transgene flow from *Bt/CpTI* rice to its nontransgenic counterparts planted at close spacing. New Phytologist 168: 559–566. 16313639 10.1111/j.1469-8137.2005.01539.x

[23] Loureiro I, Escorial M, González Á, Chueca M (2012) Pollen-mediated gene flow in wheat (Triticum aestivum L.) in a semiarid field environment in Spain. Transgenic Research 21: 1329–1339. 10.1007/s11248-012-9619-x 22615061

[24] Lavigne C, Klein EK, Vallée P, Pierre J, Godelle B, Renard M (1998) A pollen-dispersal experiment with transgenic oilseed rape: estimation of the average pollen dispersal of an individual plant within a field. Theoretical and Applied Genetics 96: 886–896.

[25] Yao KM, Hu N, Chen WL, Li RZ, Yuan QH, Wang F, et al (2008) Establishment of a rice transgene flow model for predicting maximum distances of gene flow in Southern China. New Phytologist 180: 217–228. 10.1111/j.1469-8137.2008.02555.x 18643943

[26] Rong J, Song ZP, de Jong TJ, Zhang XS, Sun SG, Xu X, et al (2010) Modelling pollen-mediated gene flow in rice: risk assessment and management of transgene escape. Plant Biotechnology Journal 8: 452–464. 10.1111/j.1467-7652.2009.00488.x 20132516

[27] Klein EK, Lavigne C, Foueillassar X, Gouyon PH, Larédo C (2003) Corn pollen dispersal: quasi-mechanistic models and field experiments. Ecological Monographs 73: 131–150.

[28] Gustafson DI, Horak MJ, Rempel CB, Metz SG, Gigax DR, Hucl P (2005) An empirical model for pollen-mediated gene flow in wheat. Crop Science 45: 1286–1294.

[29] Snäll T, O’Hara RB, Arjas E (2007) A mathematical and statistical framework for modelling dispersal. Oikos 116: 1037–1050.

[30] Di-Giovanni F, Beckett PM, Flenley JR (1989) Modelling of dispersion and deposition of tree pollen within a forest canopy. Grana 28: 129–139.

[31] Giddings G (2000) Modelling the spread of pollen from *Lolium perenne*: The implications for the release of wind-pollinated transgenics. Theoretical and Applied Genetics 100: 971–974.

[32] Beckie HJ, Hall LM (2008) Simple to complex: Modelling crop pollen-mediated gene flow. Plant Science 175: 615–628.

[33] Baker J, Preston C (2003) Predicting the spread of herbicide resistance in Australian canola fields. Transgenic Research 12: 731–737. 14713202 10.1023/b:trag.0000005147.04075.62

[34] Manasse RS (1992) Ecological risks of transgenic plants: effects of spatial dispersion on gene flow. Ecological Applications 2: 431–438. 27759266 10.2307/1941878

[35] Loos C, Seppelt R, Meier-Bethke S, Schiemann J, Richter O (2003) Spatially explicit modelling of transgenic maize pollen dispersal and cross-pollination. Journal of Theoretical Biology 225: 241–255. 14575658 10.1016/s0022-5193(03)00243-1

[36] Katul GG, Porporato A, Nathan R, Siqueira M, Soons MB, Poggi D, et al (2005) Mechanistic analytical models for long-distance seed dispersal by wind. The American Naturalist 166: 368–381. 16224691 10.1086/432589

[37] Soons MB, Heil GW, Nathan R, Katul GG (2004) Determinants of long-distance seed dispersal by wind in grasslands. Ecology 85: 3056–3068.

[38] Jackson ST, Lyford ME (1999) Pollen dispersal models in quaternary plant ecology: assumptions, parameters, and prescriptions. Botanical Review 65: 39–75.

[39] Duffin KI, Bunting MJ (2008) Relative pollen productivity and fall speed estimates for southern African savanna taxa. Vegetation History and Archaeobotany 17: 507−525.

[40] Lemmon EW, Jacobsen RT (2004) Viscosity and thermal conductivity equations for nitrogen, oxygen, argon, and air. International Journal of Thermophysics 25: 21–69.

[41] Wang F, Yuan QH, Shi L, Qian Q, Liu WG, Kuang BG, et al (2006) A large-scale field study of transgene flow from cultivated rice (Oryza sativa) to common wild rice (O. rufipogon) and barnyard grass (Echinochloa crusgalli). Plant Biotechnology Journal 4: 667–676. 17309736 10.1111/j.1467-7652.2006.00210.x

[42] Matus-Cádiz MA, Hucl P, Horak MJ, Blomquist LK (2004) Gene flow in wheat at the field scale. Crop Science 44: 718–727.

[43] Zhang K, Li Y, Lian L (2011) Pollen-mediated transgene flow in maize grown in the Huang-huai-hai region in China. Journal of Agricultural Science 149: 205−216.

[44] Krato C, Petersen J (2011) Gene flow between imidazolinone-tolerant and -susceptible winter oilseed rape varieties. Weed Research 52: 187–196.

[45] Séguin-Swartz G, Beckie HJ, Warwick SI, Roslinsky V, Nettleton JA, Johnson EN, et al (2013) Pollen-mediated gene flow between glyphosate-resistant *Brassica napus* canola and *B*. *juncea* and *B*. *carinata* mustard crops under large-scale field conditions in Saskatchewan. Canadian Journal of Plant Science 93: 1083–1087.

[46] Song ZP, Lu B-R, Chen JK (2004) Pollen flow of cultivated rice measured under experimental conditions. Biodiversity and Conservation 13: 579–590.

[47] Jiang WM (1993) Simulation of air pollution under ideal conditions In: Jiang WM, Sun JN, Cao WJ, Jiang RB, eds. Introduction to air pollution meteorology. Beijing, China: China Meteorological Press, 53–200.

[48] Aylor DE (2002) Settling speed of corn (Zea mays) pollen. Journal of Aerosol Science 33: 1601−1607.

[49] Chaturvedi M, Datta Κ, Nair PKK (1998) Pollen morphology of Oryza (Poaceae). Grana 37: 79–86.

[50] Goss JA (1968) Development, physiology, and biochemistry of corn and wheat pollen. The Botanical Review 34: 333−359.

[51] Storme ND, Zamariola L, Mau M, Sharbel TF, Geelen D (2013) Volume-based pollen size analysis: an advanced method to assess somatic and gametophytic ploidy in flowering plants Plant Reproduction 26: 65−81. Oka HI (1988) Origin of cultivated rice. Japan Scientific Societies Press, Tokyo 10.1007/s00497-012-0209-0 23686220

[52] Oka HI (1988) Origin of cultivated rice. Japan Scientific Societies Press, Tokyo.

[53] Labana KS, Banga SS (1984) Floral biology in Indian mustard (Brassica juncea (L.) coss) Genetica Agraria 38: 131−138.

[54] Chauhan YS, Kumar K, Ram B (1987) Extent of outcrossing in Indian mustard (Brassica juncea L. Czern & Coss). Eucarpia Cruciferae Newsletter 12:44.

[55] Rakow G, Woods DL (1987) Outcrossing in rape and mustard under Saskatchewan prairie conditions. Canadian Journal of Plant Science 67: 147−151.

[56] Chhikara RS, Folks JL (1989) The inverse Gaussian distribution: theory, methodology and application. New York: Marcel Dekker.

[57] Tackenberg O, Poschlod P, Kahmen S (2003) Dandelion seed dispersal: the horizontal wind speed does not matter for long-distance dispersal—it is updraft. Plant Biology 5: 451–454.

[58] Tackenberg O (2003) Modeling long-distance dispersal of plant diaspores by wind. Ecological Monographs 73: 173–189.

[59] Waines JG, Hegde SG (2003) Intraspecific gene flow in bread wheat as affected by reproductive biology and pollination ecology of wheat flowers. Crop Science 43: 451–463.

[60] Langhof M, Hommel B, Hüsken A, Njontie C, Schiemann J, Wehling P, et al (2010) Coexistence in maize: isolation distance in dependence on conventional maize field depth and separate edge harvest. Crop Science 50: 1496–1508.

[61] Hucl P, Matus-Cádiz M (2001) Isolation distances for minimizing out-crossing in spring wheat. Crop Science 41: 1348–1351.

[62] Song ZP, Lu B-R, Chen JK (2001) A study of pollen viability and longevity in *Oryza rufipogon*, *O*. *sativa*, and their hybrids. International Rice Research Newsletter 26: 31–32.

[63] Luna S, Figueroa J, Baltazar B, Gomez R, Townsend R, Schoper JB (2001) Maize pollen longevity and distance isolation requirements for effective pollen control. Crop Science 41: 1551–1557.

[64] Jia SR, Wang F, Shi L, Yuan QH, Liu WG, Liao YL, et al (2007) Transgene flow to hybrid rice and its male-sterile lines. Transgenic Research 16: 491–501. 17443417 10.1007/s11248-006-9037-z

[65] Lu B-R, Snow AA (2005) Gene flow from genetically modified rice and its environmental consequences. BioScience 55: 669–678.

[66] Messeguer J (2003) Gene flow assessment in transgenic plants. Plant Cell, Tissue and Organ Culture 73: 201–212.

[67] Saeglitz C, Pohl M, Bartsch D (2003) Monitoring gene flow from transgenic sugar beet using cytoplasmic male-sterile bait plants. Molecular Ecology 9: 2035–2040. 10.1046/j.1365-294x.2000.01109.x 11123616

[68] Devos Y, Reheul D, De Schrijver A (2005) The co-existence between transgenic and non-transgenic maize in the European Union: a focus on pollen flow and cross-fertilization. Environmental Biosafety Research 4: 71–87. 16402663 10.1051/ebr:2005013

[69] Devos Y, Demont M, Dillen K, Reheul D, Kaiser M, Sanvido O (2009) Coexistence of genetically modified (GM) and non-GM crops in the European Union. A review. Agronomy for Sustainable Development 29: 11–30.

[70] Ellstrand NC (2014) Is gene flow the most important evolutionary force in plants? American Journal of Botany 101: 737–753. 24752890 10.3732/ajb.1400024

[71] Ellstrand NC, Schierenbeck KA (2000) Hybridization as a stimulus for the evolution of invasiveness in plants? Proceedings of the National Academy of Sciences 97: 7043–7050. 10.1073/pnas.97.13.7043 PMC34382 10860969

